# Comparison on chemical compositions and antioxidant capacities of the green, oolong, and red tea from blueberry leaves

**DOI:** 10.1002/fsn3.1455

**Published:** 2020-02-13

**Authors:** Zhi Chai, Liangliang Tian, Hong Yu, Liangcong Zhang, Qilong Zeng, Han Wu, Zheng Yan, Dajing Li, Ruth Paulina Hutabarat, Wuyang Huang

**Affiliations:** ^1^ Institute of Agro‐Product Processing Jiangsu Academy of Agricultural Sciences Nanjing PR China; ^2^ Institute of Botany Jiangsu Province and Chinese Academy of Sciences Nanjing PR China; ^3^ Institute of Translational Medicine & Medical College Yangzhou University Yangzhou PR China; ^4^ Jiangsu Key Laboratory for Horticultural Crop Genetic Improvement Jiangsu Academy of Agricultural Sciences Nanjing PR China; ^5^ School of Food and Biological Engineering Jiangsu University Zhenjiang PR China

**Keywords:** Antioxidant capacities, Blueberry leaves, Chemical composition, Fermentation, Tea

## Abstract

Blueberry leaves, by‐products of the blueberry industry, could be explored as source of functional foods, such as teas. Three different types of tea, including nonfermented green tea, semifermented oolong tea, and fully fermented red tea from blueberry leaves, were investigated on their chemical compositions and antioxidant capacities here. The contents of individual amino acids in three types varied, while the total amounts retained constant. A total of 167 volatiles were detected with alcohols, alkenes, and aldehydes as the dominant. More volatiles produced in the fermented teas. The total phenolic/flavonoid contents were highest in the green tea and decreased significantly in the oolong and red teas, correlating inversely with the fermentation degree. The highest levels of representative phenolics, that is, phenolic acids and flavonol glycosides, contributed to the strongest antioxidant capacity in the green tea. These indicated that blueberry leaves provided promising and prospective potential to develop new teas beneficial for health.

## Graphical abstract

Blueberry leaf teas exhibited considerable antioxidant activities, effected by their phenolic contents depending on different fermentation degrees.

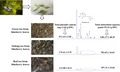

## 1 INTRODUCTION

Blueberries (*Vaccinium* spp.), generally regarded as an excellent source of dietary antioxidant, are one kind of popular and healthy fruits worldwide (Wang et al., 2015). Because of their appealing taste and attractive biological functions, blueberries have received a great deal of attention on their active constituents (Huang et al., 2018). Compared with extensively studied fruits, blueberry leaves are usually ignored and discarded as the by‐products of blueberry industry. Indeed, blueberry leaves contained high levels of nutrients and phytochemicals, especially even more phenolics than the fruits, which could also serve as an effective antioxidant resource or functional food ingredient (Ehlenfeldt & Prior, 2001). The application of the waste blueberry leaves would be of considerable interests in the related industry; however, the exploration of blueberry leaves is limited.

Tea, normally produced from the leaves of the economic plant *Camellia sinensis* L, is the second most widely consumed beverage in the world for its satisfactory sensory experience and multiple health‐promoting properties (Hajiaghaalipour, Kanthimathi, Sanusi, & Rajarajeswaran, 2015). The organoleptic properties of tea are related to a combination of various compounds, including catechins for bitterness, polyphenols for astringency, amino acids for freshness, and volatiles for aromas, which contribute to the unique and characteristic tastes, flavors, and aromas of tea (Chen, Yang, Lee, Wu, & Tzen, 2014). Meanwhile, some natural phytochemicals, especially phenolic constituents, have been revealed to be responsible for the multiple health‐promoting properties of teas (Dias et al., 2014). Sharing similar chemical components and bioactivities with the leaves of *C. sinensis*, blueberry leaves offer great potential to be further explored as one kind of new teas.

Manufacturers had made blueberry leaves available in the form as “herbal tea” (Gallaher, Gallaher, Marshall, & Marshall, 2006); however, they were actually the simple water infusions of the leaves without any conventional tea processing steps. The main types of tea are classified into green, white, yellow, oolong, red, and pu‐erh teas based on different manufacturing processes (Yi et al., 2015). Among them, the most well‐known and commonly found in the market are unfermented green tea, semifermented oolong tea, and fully fermented red tea. Lacking the relevant study, the exploration of different types of tea from blueberry leaves is promising, and the chemical composition's changes during the manufacturing process of teas are worth well elucidating.

In the present study, three different types of tea samples, including minimally, partially, and maximally oxidized blueberry leaf teas, were attempted to produce, respectively, according to the making process of the green, oolong, and red tea from *C. sinensis*. These three types of teas were thus named as green tea (GT), oolong tea (OT), and red tea (RT) from blueberry leaves hereafter. The changes of main chemical compositions and antioxidant capacities during the processing of different blueberry leaf teas were evaluated and compared. Various profiles, such as amino acids, volatile aroma components, and phenolic compounds/flavonoids/proanthocyanidin, were further investigated on the development of tea quality. Meanwhile, the antioxidant capacities in vitro of blueberry leaf teas were comprehensively assessed through four common antioxidant activity methods. This study would contribute to current knowledge on the comprehensive utilization of the waste blueberry leaves as potential valuable resource, and also the systematically development of novel tea varieties.

## 2 MATERIALS AND METHODS

### 2.1 Plant materials and tea sample preparation

Fresh leaves of Garden Blue rabbiteye blueberry (*Vaccinium ashei*) were collected from the orchards of Nanjing Botanical Garden Memorial Sun Yat‐Sen, Jiangsu, China, in August 2017. The shoots with one bud and several leaves were handpicked from the top of the newly grown branches and then brought back to the laboratory immediately for tea preparation. Three different types of tea samples were manufactured, including nonfermented GT, semifermented OT, and fully fermented RT. The manufacturing process of the GT samples was on the basis of corresponding traditional tea processing method of *C. sinensis*. The blueberry leaves were fixed by microwave at 500 W for 60 s with leaf quantity of 2 g/cm^2^ after withering at 30°C and a relative humidity (RH) of 60% for 3 hr. Then, the leaves were tossed and rolled by hand gently for 30 min before they were dried at 70°C through hot air to a final moisture content of less than 4%. For the OT samples, the blueberry leaves were turned over at 25°C and a RH of 80% for 5 min using a rotary shaker for five times with 40‐min intervals after withering. Afterward, the fermented samples were fixed by microwave, rolled at room temperature, and dried at 70°C to produce the OT. Similarly, the blueberry leaf RT samples underwent the process of withering, rolling, fermentation, and drying. Fermentation was performed at 28–30°C and a RH of 80% for 15–20 hr, and then terminated by hot air drying to a moisture content of less than 4%. The dried tea samples were ground to fine powder by a pulverizer and sifted through a 40‐mesh sieve. The powder was stored at −20°C in a refrigerator for subsequent analysis.

### 2.2 Chemicals and reagents

Formic acid, 2,2‐azinobis (3‐ethylbenzothiazoline‐6‐sulfonic acid) diammonium salt (ABTS), and 2,2‐diphenyl‐1‐picrylhydrazyl (DPPH) were obtained from Sigma‐Aldrich Chemical Co. (St. Louis, MO, USA). Trolox (6‐hydroxy‐2,5,7,8‐tetramethylchromane‐2‐carboxylic acid) was purchased from Acros Organics (Morris Plains, NJ, USA). Fluorescein disodium was purchased from Chemical Industrial of East China Normal University (Shanghai, China). Gallic acid, rutin, catechin, 2,2’‐azobis(2‐methylpropionamide)‐dihydrochloride (AAPH), and Folin‐Ciocalteu's reagent were obtained from J&K Chemical Ltd. (Beijing, China). The HPLC standards, including 3‐caffeoylquinic acid, 4‐caffeoylquinic acid, 5‐caffeoylquinic acid, 3,5‐dicaffeoylquinic acid, 4,5‐dicaffeoylquinic acid, kaempferol‐3‐O‐glucoside, quercetin, quercetin‐3‐O‐galactoside, quercetin‐3‐O‐rutinoside, and quercetin‐3‐O‐arabinoside, were bought from Yuanye Chemical Ltd. (Shanghai, China). Methanol and acetonitrile (chromatographic grade) were obtained from TEDIA (Ohio, USA). The purity of the standards and solvents used for HPLC was verified > 95%. All the other chemicals and reagents were of analytical grade.

### 2.3 Preparation of extracts

The extracts were prepared according to the reported method with some modifications (Yang et al., 2009). The dried powder of different tea samples was extracted by 85% methanol solution containing 0.5% formic acid for three times. The extracts were stored at 4°C prior to further assay.

### 2.4 Amino acid analysis

The amino acid analysis was performed on an Agilent 1100 Series HPLC system (Agilent Technologies, USA) equipped with a binary pump, a DAD and a fluorescence detector (FLD). The separation was carried out at 40°C using a ZORBAX Eclipse AAA column (4.6 × 150 mm, 3.5 μm). Mobile phase A was 40 mM NaH2PO4 solution (pH = 7.8), and mobile phase B was acetonitrile/methanol/H2O (45:45:10, v/v/v). The gradient elution program was as follows: 0% B (from 0 to 1.9 min), 0% to 57% B (from 1.9 to 18.1 min), 57% to 100% B (from 18.1 to 18.6 min), 100% B (from 18.6 to 22.3 min), 100% to 0% B (from 22.3 to 23.2 min), and 0% B (from 23.2 to 26 min).

### 2.5 Gas chromatography–mass spectrometer (GC‐MS)

Separation and identification of the volatiles were conducted on an Agilent 7890A‐5975C GC‐MS system (Agilent Technologies) equipped with a DB‐WAX capillary column (0.25 mm × 0.25 μm × 50 m). Helium was used as the carrier gas, with a rate of 1.5 ml/min. The oven temperature was programmed from 50°C (retained for 1 min) to 100°C (retained for 5 min) at a rate of 5°C/min, ramped up to 140°C (retained for 10 min) at a rate of 4°C/min, raised to 180°C (retained for 10 min) at a rate of 4°C/min, and finally increased to 250°C (retained for 5 min) at a rate of 4°C/min. Samples were injected into the GC injection port held at 250°C. MS was operated in full scan mode (mass range, m/z 50–550) with ionization voltage at 70 eV, and ion source temperature at 230°C. A library search was carried out using the in‐house database of NIST11.L.

### 2.6 Composition analysis

The Folin–Ciocalteu colorimetric method was employed to determine the total phenolic content (TPC) according to the previous report (Huang, Zhang, Liu, & Li, 2012). TPC values were expressed as gallic acid equivalent (GAE), that is, mg GAE/g dry weight (DW). The total flavonoid content (TFC) was determined by a colorimetric method. The TFC values were expressed as rutin equivalents (RTE), that is, mg RTE/g DW. The vanillin–hydrochloric acid method was conducted for the determination of the proanthocyanidin content (PAC) (Nakamura, Tsuji, & Tonogai, 2003). The PAC was expressed as catechin equivalents (CTE), that is, mg CTE/g DW.

### 2.7 *Assay of antioxidant activity *in vitro

The scavenging activity for DPPH radical was estimated through spectrophotometric method (Xiao et al., 2014). The scavenging activity for ABTS radical cation (ABTSˑ^+^) was performed according to the previous method (Arts, Dallinga, Voss, Haenen, & Bast, 2004). The DPPH and ABTS results were expressed in terms of trolox equivalent antioxidant capacity (TEAC), that is, µmol TEAC/g DW. The ferric reducing antioxidant power (FRAP) assay was conducted based on the method of Razak, Rashid, Jamaluddin, Sharifudin, and Long (2015). The FRAP results were expressed as Fe (II) equivalent antioxidant capacity (FEAC), that is, μmol Fe (II)/g DW. The oxygen radical absorbance capacity (ORAC) assay was carried out to assess the total antioxidant activity (Li, Huang, Wang, & Liu, 2013). The fluorescence was recorded by a LB 941 TriStar Microplate Reader (Berthold Technologies, Bad Wildbad, Germany). The final ORAC value was also expressed as TEAC.

### 2.8 High‐performance liquid chromatographic (HPLC) analysis

The samples were subjected to an Agilent 1100 HPLC system (Agilent Technologies, USA) equipped with a binary pump, a diode‐array detector (DAD), and an end‐capped reverse‐phase Zorbax SB‐C18 column (250 mm × 4.6 mm, 5 μm). The injection volume was 10 μL. Mobile phase A and phase B were 1% formic acid (TFA) and 100% methanol, respectively, at a flow rate of 0.6 ml/min. The elution gradient was as follows: 10% to 60% B (from 0 to 25 min), 60% to 80% B (from 25 to 40 min), and 80% to 10% B (from 40 to 45 min). The phenolic compounds were detected at 280, 320, and 360 nm, and identified by comparing both retention times (t_R_) and ultraviolet (UV) spectra with authentic standards. Ten phenolics were further quantified, expressed as milligram of each compound per gram of the dried weight, that is, mg/g DW.

### 2.9 HPLC‐ESI‐MS analysis

HPLC‐ESI‐MS analysis was carried out on an Agilent 1100 HPLC/MS(SL) system equipped with a UV detector and a LC‐MSD Trap VL ion‐trap mass spectrometer (MS) via an electrospray ionization (ESI) interface (Agilent Technologies). HPLC separation conditions were the same as described above. For MS conditions, the ESI capillary voltage was 3.0 kV in negative ion (NI) mode with the capillary temperature at 350°C. A nebulizing gas of 1.5 L/min and a drying gas of 10 L/min were applied for ionization using nitrogen (N_2_). ESI was performed with the scan range at m/z 150–1000.

### 2.10 Statistical analysis

All determinations were conducted in triplicate, and the results were calculated as mean value ± standard deviation (*SD*). The fluorescence decay curve and figure data in ORAC assay were obtained using GraphPad Prism Version 5.02 (GraphPad Software, Inc., CA, USA). One‐way analysis of variance (ANOVA) with Tukey's *post hoc* test was used to determine statistical differences among 3 tea groups and the tested indices. Differences were considered significant with *p* < .05.

## 3 RESULTS AND DISCUSSION

### 3.1 Amino acid content in blueberry leaf teas

The amino acid contents in blueberry leaf GT, OT, and RT are listed in Table 1. The GT and RT showed the high total amounts of amino acid at 28.734 and 28.535 mg/g DW, respectively, followed by the OT at 27.608 mg/g. The teas from blueberry leaves here revealed an elevated total amounts of amino acid than existing commercial teas from leaves of the plant *C. sinensis* L, which was reported previously to range from 13.2 to 20.2 mg/g (Horanni & Engelhardt, 2013).

**Table 1:** Content of amino acids (mg/g on dry weight basis) in the different tea samples from blueberry leaves

Amino acid	GT	OT	RT
Asp	1.918	1.895	1.986
Glu	1.253	1.390	1.346
Ser	1.253	1.271	1.283
His	0.251	0.217	0.219
Gly	1.701	1.521	1.613
Thr	0.682	0.662	0.706
Arg	1.859	1.617	1.802
Ala	2.181	2.178	2.305
Tyr	1.195	1.159	1.225
Cys	0.000	0.000	0.000
Val	2.539	2.291	2.593
Met	0.485	0.307	0.300
Phe	1.741	1.829	1.914
Ile	1.704	1.655	1.795
Leu	2.953	2.840	3.026
Lys	0.882	0.887	0.874
Pro	6.137	5.889	5.548
Total	28.734	27.608	28.535

The contents of individual amino acids varied in the GT, OT, and RT samples. Notably, proline (Pro) was the most abundant amino acid among the three teas. Its content in the GT (6.137 mg/g) was higher than that of the OT and RT (5.889 and 5.548 mg/g, respectively), indicating an inverse correlation with the degree of fermentation. Except for Pro, aspartic acid (Asp), glutamic acid (Glu), serine (Ser), glycine (Gly), arginine (Arg), alanine (Ala), tyrosine (Tyr), valine (Val), phenylalanine (Phe), leucine (Leu), and isoleucine (Ile) were the major amino acids, accounting for approximately 70.64%–73.20% of the total amino acids and 1.96%–2.09% of dry weight. It has been reported that most of the above amino acids contribute to the sweet or bitter taste of tea (Kirimura, Shimizu, Kimizuka, Ninomiya, & Katsuya, 1969).

The content and composition of amino acids were recognized to be closely related to the sensory property and consequently the quality of tea (Alcazar et al., 2007). Some amino acids are the main contributors for the umami taste of tea, especially Glu (Kaneko, Kumazawa, Masuda, Henze, & Hofmann, 2006). The content of Glu, representing 1.253 mg/g dry weight, was found to be the lowest in the GT. The OT and RT contained higher Glu contents at 1.390 and 1.346 mg/g, respectively. This demonstrated that fermentation process could increase the intensity of umami taste. In addition, the Ser and Phe levels in the OT and RT were higher than that in the GT. On the contrary, higher amounts of histidine (His), Gly, Arg, and methionine (Met) were present in the nonfermented GT. A clear corresponding relationship between their contents and fermentation process could be observed. During the fermented process, protein breakdown, polymerization, or transformation could occur, which led to great changes on the content and composition of amino acids (Horanni & Engelhardt, 2013).

### 3.2 Volatile compositions of blueberry leaf teas

The volatile compositions were found to vary significantly in the three different tea types, both qualitatively and quantitatively. A total of 167 volatile compounds were structurally identified in the three tea samples from blueberry leaves, 98 of which with integration area percentage > 0.2% and match quality > 40 are listed in Table 2. The number of volatile compounds in the GT was 105, which increased to 116 in the OT and 133 in the RT. Among the identified components, 71 volatiles were found in all three samples simultaneously, and 29 were detected in the semifermented OT and fully fermented RT, as well as 25 only in the RT, implying new volatiles were generated during the fermentation process. In addition, 21 volatiles were detected only in the GT, which might be interpreted as aroma precursors and undergone degradation or transformation during fermentation. Meanwhile, 11 volatile compounds were only detected in the OT, which were speculated to be served as intermediate metabolites formed accompanying the enzymatic oxidation of tea fermentation.

**Table 2:** Main volatile composition in the different tea samples from blueberry leaves

Library/ID	t_R_ (min)	Area percent (%)		
		GT	OT	RT
Alcohols				
2‐Heptanol	11.7805			0.3990
1‐Decanol	12.1955	0.7020		
cis‐alpha,alpha,5‐Trimethyl−5‐vinyltetrahydrofuran−2‐methanol	16.5279	0.4126	0.7125	0.8095
1‐Octen−3‐ol	16.6513	0.6194	0.3733	0.3852
trans‐Linalool oxide	17.6971	0.4070	0.6390	0.6925
2‐Methylene cyclopentanol	20.2868		0.4124	
(±)−3,7‐Dimethyl−1,6‐octadien−3‐ol	20.5819	11.1936	3.6398	2.5011
1‐Octanol	20.9462	0.7941	0.5770	0.5924
(R)−4‐Methyl−1‐(1‐methylethyl)−3‐cyclohexen−1‐ol	22.6090	2.1511	0.5529	0.5467
3,7‐Dimethyl−1,5,7‐octatrien−3‐ol	22.9439	1.3569	0.7484	0.5021
5‐Methyl−2‐(1‐methylethyl)‐cyclohexanol	23.9192	0.2971	0.6597	0.5236
2‐Cyclohexen−1‐ol	24.1462	1.2450		
1‐Nonanol	24.5831	0.2617	0.4975	0.5803
alpha‐Terpineol	25.9639	7.9837	6.2882	7.6880
2‐Cyclohexen−1‐ol	27.9909	0.5337	0.4543	0.0937
(Z)−3,7‐Dimethylocta−2,6‐dien−1‐ol	30.1883	0.5524	0.2170	0.1235
Geraniol	32.6736	1.9864	1.2197	0.6502
Butylated hydroxytoluene	36.3282	0.3791	0.3623	0.3224
Phenylethyl alcohol	36.5750	0.1387	1.0605	2.6369
Maltol	39.5538		2.2743	0.8045
Aldehydes				
2‐Methylbutyraldehyde	4.4127			1.5072
Hexanal	6.4984	0.7777	0.7205	0.6563
(2E)‐Hexenal	9.3950		0.3976	1.0804
Octanal	10.8404	1.1920	0.6886	0.3455
(Z)−2‐Heptenal	12.0860	0.6862	0.7532	0.4486
Nonanal	14.2423	5.5595	3.0881	3.2285
(E)−2‐Octenal	15.9697	1.2979	1.1344	0.5986
Furfural	17.5913			0.5596
Decanal	18.6195		0.6385	0.6197
2,4‐(E,E)‐Heptadienal	18.7547	2.1454	2.3345	1.2512
Benzaldehyde	19.8945	1.0212	0.8755	1.2771
(E)−2‐Nonenal	20.1765	0.9814	0.8332	0.9998
(2E,6E)‐nona−2,6‐dienal	22.0861	0.6804	0.9900	1.0362
2,6,6‐Trimethyl−1‐cyclohexene−1‐carboxaldehyde	23.2083	0.8908	1.0427	0.9086
2,3‐Dihydro−2,2,6‐trimethylbenzaldehyde	24.0955		0.7405	0.7296
(E)−2‐Decenal	24.1528		0.7643	
Benzeneacetaldehyde	24.1718			0.4419
p‐Menth−1‐en−9‐al	25.5643	0.3720	1.0530	0.8593
Esters				
Ethyl caprylate	15.8522		0.2853	0.6188
Hexanoic acid,3‐hexen−1‐yl ester	24.3775			0.5070
Propanoic acid, 2‐methyl‐, 2,2‐dimethyl−1‐(1‐methylethyl)−1,3‐propanediyl ester	34.5580		0.9114	
1,5‐Octalactone	39.5516	0.4253		
4,4,7a‐Trimethyl−5,6,7,7a‐tetrahydrobenzofuran−2(4H)‐one	55.3177		0.5260	0.2036
Acids				
Glacial acetic acid	17.0038		1.1034	0.4574
2‐Amino−4‐methylbenzoic acid	28.6548	0.1835	0.2665	0.3339
Hexanoic acid	32.5268	0.4343	0.8241	0.5648
(E)−3‐Hexenoic acid	38.9486		0.4270	0.2278
Octanoic acid	43.2965	5.5331	9.6354	2.0701
Nonanoic acid	47.1332	0.8129	1.9604	1.0313
*n*‐Decanoic acid	51.7983	0.6663	1.0666	0.2098
Ketones				
1‐Octen−3‐one	11.3200	0.6191		
6‐Methyl−5‐hepten−2‐one	12.3680	0.8207	0.7166	0.7309
2‐Nonanone	14.1542	0.3859	1.2474	6.3884
3,5‐Octadien−2‐one	19.6888	0.3989	0.7340	0.7591
2‐Undecanone	22.4268	0.5868	0.5197	0.9766
(E)−6‐Methyl−3,5‐heptadien−2‐one	22.5310		0.4988	0.5989
(E)−6,10‐Dimethylundeca−5,9‐dien−2‐one	33.0027	0.5925	0.9479	0.7613
beta‐Ionone	37.7970	0.2328	0.5486	0.5488
1‐(4‐tert‐Butylphenyl)propan−2‐one	37.8969		0.8207	0.622
3‐Methyl‐(cis−2‐penten−1‐yl)−2‐cyclopenten−1‐one	38.1320	0.3381	0.2682	0.1178
Alkenes				
beta‐Myrcene	7.5795	2.7224	0.5504	0.4271
2‐Carene	7.9614	0.9970	0.6664	0.2902
(+)‐Dipentene	8.3727	9.5143	3.7627	4.1295
alpha‐Phellandrene	8.8112	1.4537		
Cyclopentene	8.8310			0.4227
alpha‐Pinene	9.3517	1.7000		
gamma‐Terpinene	9.4700	2.5130	0.5523	
(Z)−3,7‐Dimethyl−1,3,6‐octatriene	9.7806	2.2330		
alpha‐Phellandrene	9.8747	0.5453		
1,3‐p‐Menthadien	10.3763	4.6552	1.4054	0.6969
2,3‐Dimethyl−1‐hexene	11.2517	0.3435	0.4534	0.1478
1,3,5,5‐Tetramethyl−1,3‐cyclohexadiene	13.5174	0.2183		
cis−2,6‐Dimethyl−2,6‐octadiene	14.6052		0.4496	
(2‐Methyl−1‐propenyl)benzene	16.0343	0.3862		0.7036
(1s)−2,6,6‐Trimethylbicyclo[3.1.1]hept−2‐ene	19.5655	0.2459	0.5220	0.5285
(1R,4S)−2,3‐Dimethylbicyclo[2.​2.1]hept−2‐ene	23.1062	0.7044		
cis,cis−1,3‐Cyclooctadiene	25.4644	0.1952	0.2853	0.3591
Anethole	31.5338	0.4660	1.0660	2.7888
Heterocyclic compounds				
Hexamethylcyclotrisiloxane	3.8817	0.2125		
Octamethylcyclotetrasiloxane	5.2234	0.3522	1.1262	1.7093
2,6,6‐Trimethyl−2‐ethenyltetrahydro−2H‐pyran	6.8275	0.7478	0.4016	0.4312
Decamethylcyclopentasiloxane	8.1906		3.3864	5.5769
2‐Pentylfuran	9.1541		0.5867	0.5402
cis−2‐(2‐Pentenyl)furan	10.9814	0.4224	0.7397	0.3942
Dodecamethylcyclohexasiloxane	13.3962	0.3319	1.8150	3.7003
3,6‐Dimethyl−2,3,3a,4,5,7a‐hexahydrobenzofuran	19.3011		0.2524	0.3181
Tetradecamethylcycloheptasiloxane	21.2282	0.2820	0.4599	0.7806
2‐Butyltetrahydrofuran	34.5597	0.3908		0.6091
Linear or aromatic hydrocarbons				
*n*‐Hexane	3.2082	2.2164	4.2192	5.7039
2,2,4,6,6‐Pentamethyl‐heptane	4.8298	0.6864	0.8094	0.8789
p‐Xylene	7.1447		0.3970	0.1758
Vinylcyclopentane	8.8472		0.4960	
o‐Cymene	10.1882	1.7021	1.0180	1.4169
*n*‐Propylbenzene	24.2717			0.8093
(E)−1,1‐Dimethyl−2‐(3‐methylbuta−1,3‐dien−1‐yl)cyclopropane	24.4774	0.3823	0.6502	0.7277
1‐Methylene−4‐(1‐methylvinyl)cyclohexane	25.2118	0.3871	0.2422	0.1543
Azulene	27.5561	0.1953		0.4300
2‐Methylnona−4,5‐diene	56.4928	0.3909	5.8132	2.1415

**Figure 1:**
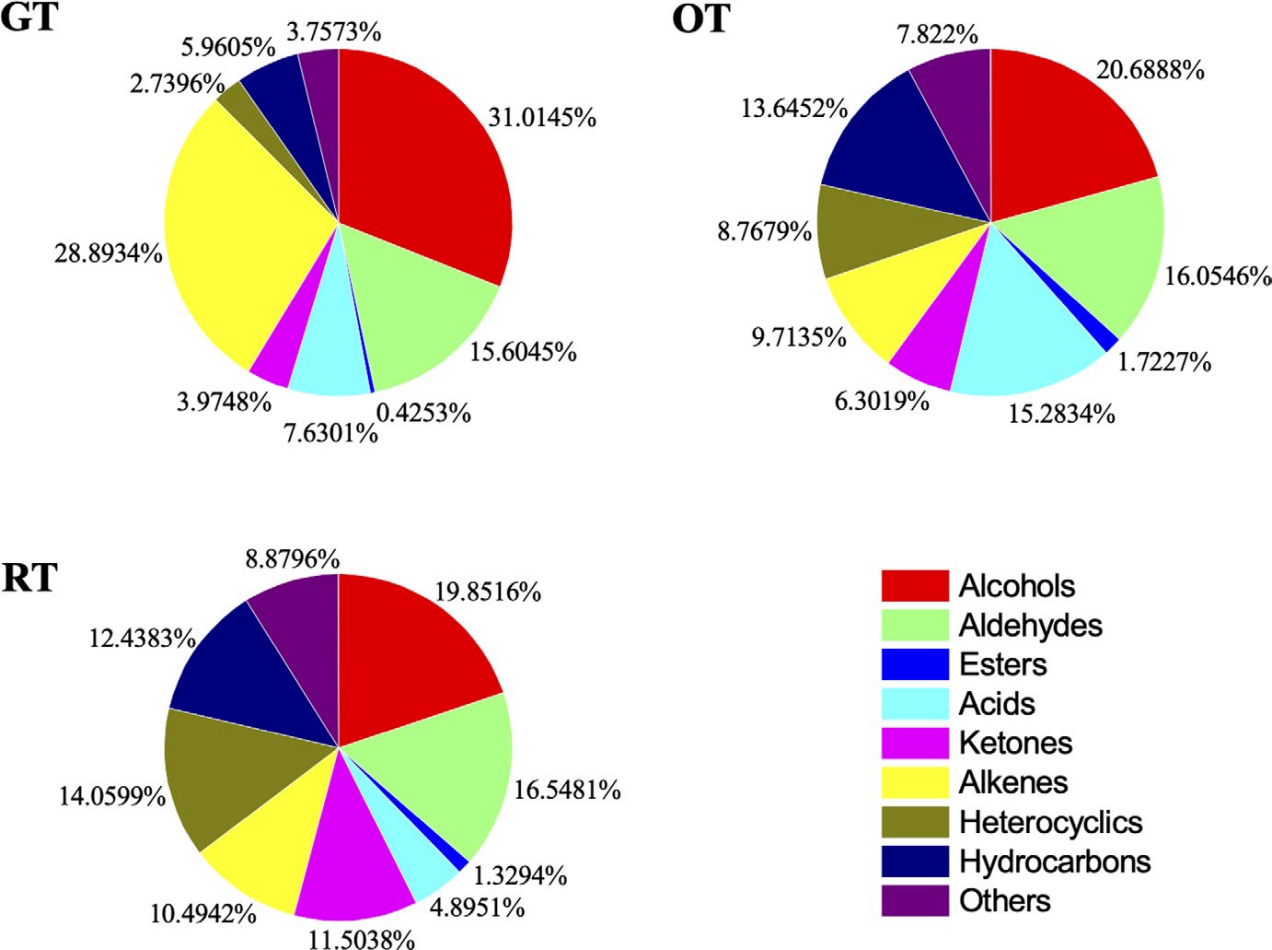
Volatile categories of different tea samples from blueberry leaves

The volatile compounds identified mainly belonged to eight chemical categories, including alcohols (20), aldehydes (18), esters (5), acids (7), ketones (10), alkenes (18), heterocyclic compounds (10), and linear or aromatic hydrocarbons (10). Briefly, alcohols (19.8516%–31.0145%), alkenes (9.7135%–28.8934%), and aldehydes (15.6045%–16.5481%) were dominant (Figure 1). In addition, the proportions of these eight chemical categories changed in different tea samples. There tended to contain the most amount of alcohols (31.0145%) and alkenes (28.8934%) in the GT. Obviously, (±)‐3,7‐dimethyl‐1,6‐octadien‐3‐ol (11.1936%) and (+)‐dipentene (9.5143%) were the most abundant compounds in the GT, which might be the major contributors for the green tea flavor. The major components in the RT were 2‐nonanone (6.3884%), n‐hexane (5.7039%), and decamethylcyclopentasiloxane (5.5769%). The aldehyde substances retained substantially in the three different tea samples after fermentation, with the proportion ranging from 15.6045% to 16.5481%. Alpha‐terpineol (6.2882%–7.9837%) was also observed as a main volatile with relatively consistent contents in all the tea samples. The proportions of alcohols and alkenes were observed to decrease in the OT and RT in accordance with the degree of fermentation process. On the contrary, the proportion of ketones, esters, heterocyclic compounds, and hydrocarbons increased in the OT and RT. This revealed that these four chemical categories were generated and released gradually during fermentation, which were accountable for the fermented tea flavor.

Different volatiles contributed to different flavors. For example, beta‐ionone, linalool, and benzeneacetaldehyde were contributors for sweet floral aroma and honey‐like flavor, respectively (Ho, Zheng, & Li, 2015). The contents of these compounds showed an increasing trend from the GT to the fermented OT and RT, which demonstrated that the fermentation promote the formation of delightful flavor. On the contrary, the nonanal, imparting inferior aroma, reduced significantly in the fermented tea samples (Xu et al., 2018).

There was a great difference on the volatile compositions of the teas from blueberry leaves in comparison with that of the commercial teas. Indole, alpha‐farnesene, and (E)‐nerolidol were reported as the characteristic volatiles and major contributors for the aroma of teas made from the leaves of *C. sinensis* L (Gui et al., 2015). However, all these components were found at trace level in the blueberry leaf teas in this study due to species differences.

### 3.3 TPC, TFC, and PAC in blueberry leaf teas

**Figure 2:**
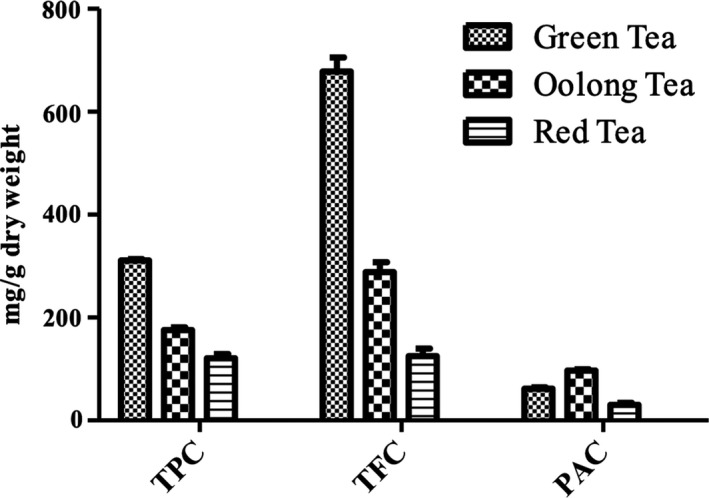
Total phenolic content (TPC), total flavonoid content (TFC), and proanthocyanidin content (PAC) of the different tea samples from blueberry leaves

As shown in Figure 2, the levels of TPC, TFC, and PAC showed dramatic variations in different teas from blueberry leaves. The highest TPC and TFC values, that is, 311.14 ± 2.28 mg GAE/g DW and 678.30 ± 27.11 mg RTE/g DW, respectively, were present in the sample of GT. Both the TPC and TFC levels decreased significantly (*p* < .001) in the OT (175.41 ± 5.75 mg GAE/g DW and 288.19 ± 19.61 mg RTE/g DW, respectively) and the RT (121.00 ± 8.12 mg GAE/g DW and 125.10 ± 14.48 mg RTE/g DW, respectively), exhibiting a fermentation degree‐dependent manner.

As the GT was derived directly from fresh blueberry leaves without additional process, its TPC and TFC values were comparable to those of the fresh leaves (357.27 ± 13.07 mg GAE/g DW and 881.55 ± 39.30 mg RTE/g DW, respectively) (our unpublished data). The minor loss might be attributed to the degradation during the withering process. The fermentation process played an important role in the change of polyphenols and flavonoids in blueberry leaf teas. The levels of TPC and TFC both decreased gradually in semifermented OT and fully fermented RT, that is, with the elevated degree of fermentation. Similar result was observed in the study of unfermented and fermented Sri Lankan tea (Jayasekera, Kaur, Molan, Garg, & Moughan, 2014).

For the proanthocyanidins, the OT showed the highest PAC value at 97.13 ± 2.64 mg CTE/g DW, followed by the GT at 61.77 ± 2.88 mg CTE/g DW and the RT at 30.09 ± 4.30 mg CTE/g DW. Proanthocyanidins were identified as one of the major components in the leaves of rabbiteye blueberry (Matsuo et al., 2010). In this study, all the three teas had significantly (*p* < .001) larger amounts of proanthocyanidin than the fresh blueberry leaves (27.64 ± 5.41 mg CTE/g DW) (our unpublished data). According to previous reports, the proanthocyanidins with mean polymerization degree of 3.1 from the leaves of rabbiteye blueberry could inhibit the proliferation of human T‐cell lymphotropic virus type 1‐associated cell lines (Nagahama et al., 2014), and the ones with polymerization degree of 8–9 have obvious potential to suppress the expression of hepatitis C virus RNA (Takeshita et al., 2009). Similarly, large amounts of procyanidin dimers and trimers were also detected in the leaf samples of other berry fruits, including lingonberry (*Vaccinium vitis‐idaea*), hawthorn (*Crataegus* spp.), and saskatoon (*Amelanchier alnifolia*) (Tian et al., 2018).

### 3.4 Antioxidant activity of blueberry leaf teas

**Figure 3:**
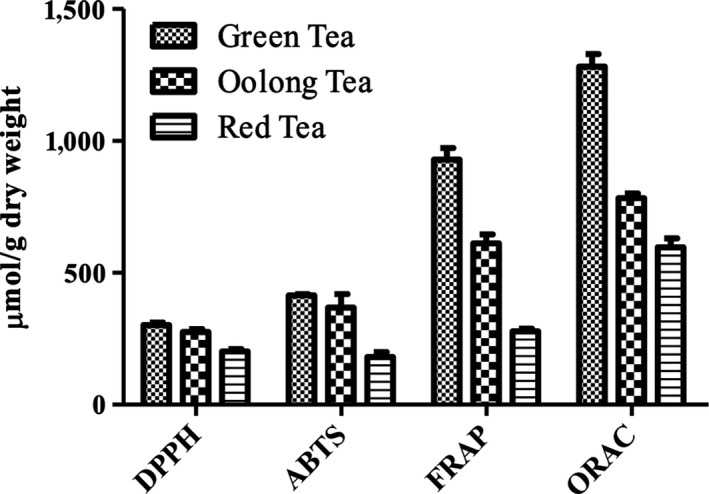
DPPH radical scavenging activity, ABTS radical scavenging activity, ferric reducing antioxidant power (FRAP), and oxygen radical absorbance capacity (ORAC) of the different tea samples from blueberry leaves

The results of DPPH, ABTS, FRAP, and ORAC assays are shown in Figure 3. All the three tea samples exhibited obvious scavenging activities on the free radicals tested in the assays, and the scavenging activities changed significantly in the different tea samples. The GT had the highest DPPH radical scavenging activity at 301.67 ± 10.33 µmol TEAC/g DW, while the OT and RT at lower values of 275.39 ± 11.11 and 201.86 ± 8.58 µmol TEAC/g DW, respectively. Similarly, the scavenging activities of ABTS free radical decreased in the following order: GT (414.54 ± 5.40 µmol TEAC/g DW)> OT (368.29 ± 50.91 µmol TEAC/g DW)> RT (181.21 ± 18.27 µmol TEAC/g DW). The FRAP value of the GT was 929.88 ± 43.11 μmol Fe (II)/g DW, which was significantly higher than that of the OT and RT (*p* < .001). This indicated that the FRAP value decreased by 34.2% and 70.1% in the OT and RT, respectively, during the fermentation process. The highest ORAC value was observed in the GT at 1,281.27 ± 48.25 μmol TEAC/g DW and decreased dramatically by 38.8% and 53.4% in the OT and GT, respectively.

Among these in vitro antioxidant models, the DPPH and FRAP assays were normally used to evaluate antioxidants in lipophilic and hydrophilic system, respectively, while the ABTS assay used in both (Lee, Seo, Lim, & Cho, 2011). The ORAC assay is a reliable technique that combines the inhibition percentage of several varieties of reactive oxygen species of biologically relevant source with time (Prior & Cao, 1999). Therefore, ORAC is largely utilized to assess the total antioxidant capacity. It could also be accounted for the results that ORAC values were much higher than the DPPH/ABTS/FRAP values.

The results in vitro indicated that the blueberry leaf GT, OT, and RT all could be potent sources of antioxidants. Their antioxidant activities decreased in the following order: GT > OT>RT, which were highly correlated with their fermentation degrees. Previous researchers had reported that there was a direct and positive relationship between the antioxidant capacity and the total phenolic content (Lin et al., 2016). Hence, phenolic compounds in the blueberry leaf teas could serve as a major contributor to their antioxidant capacity, and this was in agreement with the result of TPC measurements. The same as TPC, the antioxidant activities of different tea samples were also dependent on their corresponding fermentation degree. Fermentation process led to significantly decreased antioxidant activities. Previous studies had also reported that unfermented tea possessed higher antioxidant activity than fermented tea (Satoh, Tohyama, & Nishimura, 2005).

### 3.5 Phenolic constituents of blueberry leaf teas

Eleven phenolic compounds were differentiated and identified in blueberry leaf tea samples, ten of which were quantified (Table 3). Representative categories mainly included phenolic acids (6 caffeoylquinic acids and their derivatives) and flavonoids (specifically flavonols, i.e., 5 quercetin, kaempferol, and their glycosides). Chlorogenic acid, 3‐caffeoylquinic acid, was identified as the most abundant phenolic compound (peak 2). The peaks 1, 3, 5, 7, and 8 were identified as different derivatives of caffeoylquinic acid, that is, 5‐caffeoylquinic acid, 4‐caffeoylquinic acid, malonyl‐caffeoylquinic acid, 3,5‐dicaffeoylquinic acid, and 4,5‐dicaffeoylquinic acid, respectively. Among them, malonyl‐caffeoylquinic was not quantified due to lack of a standard. Flavonol glycosides were identified as the other major type of phenolic compounds in blueberry leaf teas, representing total amounts of 20.469 mg/g DW in the GT, 16.019 mg/g DW in the OT, and 6.774 mg/g DW in the RT, respectively. This result was consistent with previous analysis of fresh blueberry leaves, which also found that chlorogenic acid and flavonol glycosides (quercetin and kaempferol) were the major phenolic components (Oszmiański, Wojdyl̷o, Gorzelany, & Kapusta, 2011).

**Table 3:** Phenolic compounds identified in the different tea samples from blueberry leave

Peak	Compound	t_R_ (min)	[M‐H]^‐^ (m/z)	Content (mg/g DW)		
				GT	OT	RT
1	5‐caffeoylquinic acid	13.604	191, 353	1.628 ± 0.176	1.357 ± 0.024	0.332 ± 0.039
2	3‐caffeoylquinic acid	17.113	191, 353, 707	114.206 ± 6.751	44.097 ± 0.786	16.982 ± 3.584
3	4‐caffeoylquinic acid	17.829	191, 353, 707	0.019 ± 0.016	0.128 ± 0.055	0.014 ± 0.012
4	Quercetin−3‐O‐rutinoside	19.731	463, 609	3.677 ± 0.651	1.765 ± 0.557	1.279 ± 0.624
5	Malonyl‐caffeoylquinic acid	20.287	439	*n*.q.^a^	*n*.q.	*n*.q.
6	Quercetin−3‐O‐arabinoside	21.012	433	3.479 ± 0.185	0.357 ± 0.010	0.137 ± 0.021
7	3,5‐dicaffeoylquinic acid	24.411	515	2.448 ± 0.302	2.558 ± 0.324	1.002 ± 0.148
8	4,5‐dicaffeoylquinic acid	26.255	515	8.357 ± 0.885	6.182 ± 0.829	3.666 ± 0.392
9	Quercetin−3‐O‐galactoside	26.535	463	7.558 ± 0.962	8.434 ± 0.484	2.098 ± 0.434
10	Kaempferol−3‐O‐glucoside	28.503	447	4.091 ± 1.079	4.162 ± 0.839	2.302 ± 0.613
11	Quercetin	31.000	301	1.664 ± 0.538	1.301 ± 0.363	0.958 ± 0.322

a Not quantified due to lack of a standard.

**Figure 4:**
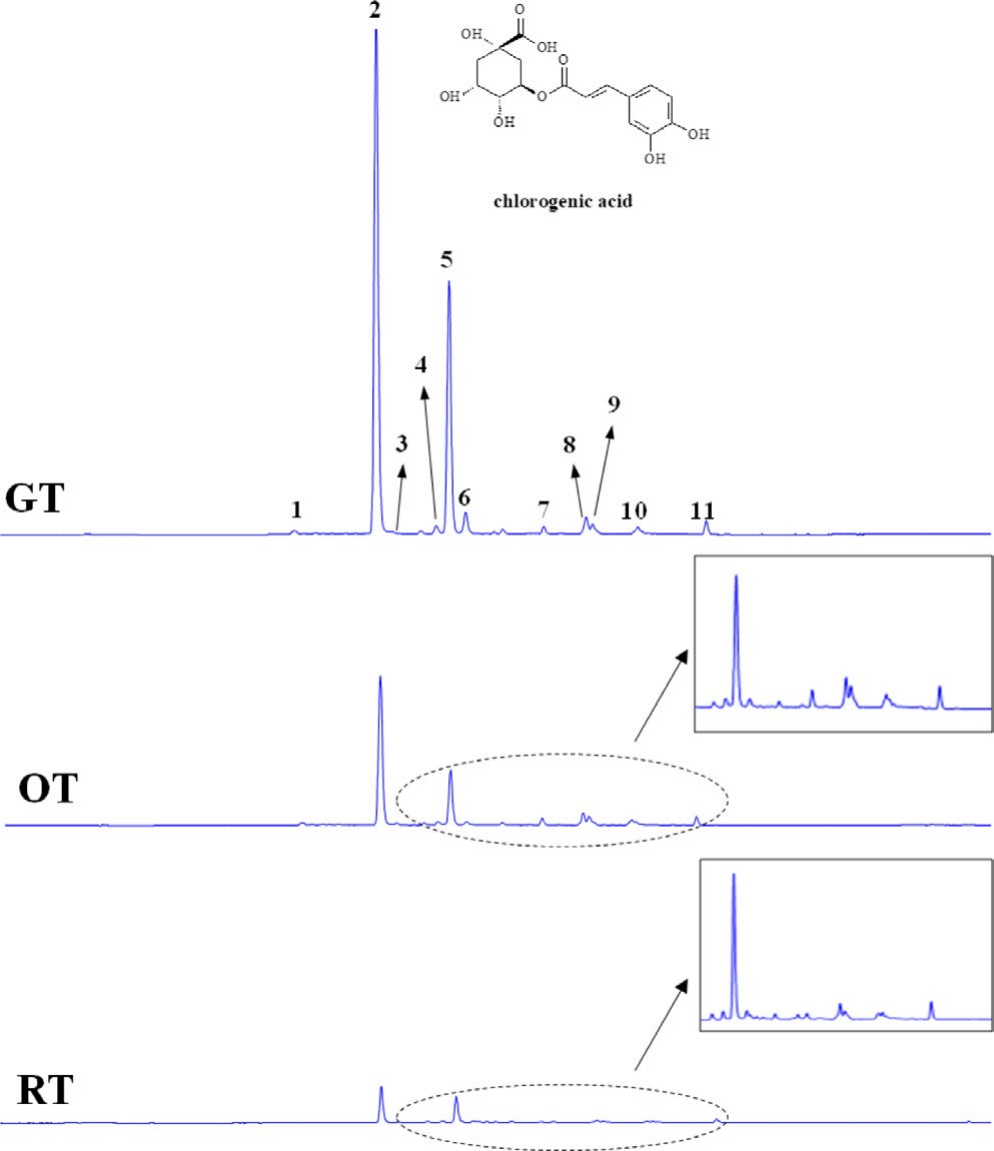
HPLC chromatographs of major phenolic compounds in the different tea samples from blueberry leaves

The typical chromatograms of the GT, OT, and RT showed visually distinct differences (Figure 4). The highest contents of most phenolic compounds were detected in the GT, for example, 114.206 ± 6.751 mg 3‐caffeoylquinic acid/g DW and 8.357 ± 0.885 mg 4,5‐dicaffeoylquinic acid/g DW, respectively. Similar to the total phenolic content measurements, a significant decrease (*p* < .001) was observed in the contents of the identified phenolic compounds with relevance to the fermentation degree of the tea samples. During the fermentation process for making oolong tea and red tea, phenolic compounds were transformed to various oxidation products in varying degrees by the endogenous enzymes, mainly polyphenol oxidase and peroxidase from fermentative microorganisms (Tan, Engelhardt, Lin, Kaiser, & Maiwald, 2017). Accompanying the oxidation and conversion of phenolics accomplished, the phenolic compounds measured consequently achieved a considerable decrease in the concentrations. Similar results were found in unfermented and fermented Sri Lankan tea (Jayasekera et al., 2014). This was also in consistence with the total phenolic content measurements.

Since the phenolics were considered as the most significant contributor to the total antioxidant capacity of tea (Ehlenfeldt & Prior, 2001), the high content of the major phenolics in the GT, specifically chlorogenic acid, could be responsible for its strong free radical scavenging activities and total antioxidant capacity. On the contrary, fermented tea, that is, the OT and RT, with lower levels in both total and individual content of phenolic compounds, showed relatively decreased antioxidant capacities, which was in agreement with previous studies (Satoh et al., 2005). In addition, phenolic compounds were also particularly important to the color, taste (especially astringency), and overall quality of teas (Shih et al., 2017). Different content of phenolic compounds could be accounted for various sensory characteristics of teas. For example, the high level of the flavonol glucosides in the GT was reported to induce a silky, mouth‐drying, and mouth‐coating sensation (Wu, Xu, Héritier, & Andlauer, 2012).

Similar to the volatile compositions and amino acid contents, the blueberry leaf teas also showed great differences on the phenolic constituents in comparison with the commercial teas made from the leaves of *C. sinensis* L, which mainly included catechins and their gallates in green tea, and dimeric theaflavins and polymeric thearubigins in fermented oolong and red teas (Menet, Sang, Yang, Ho, & Rosen, 2004). However, these components were not found in the blueberry leaf teas in this study. Instead, large amount of phenolic acids and flavonols were detected. Plenty of epidemiological studies had indicated that phenolics, such as chlorogenic acid, or flavonols like quercetin, were considered having health‐promoting or chronic disease‐preventing properties (Cai, Sun, Xing, Luo, & Corke, 2006). These functional phenolic compounds rich in blueberry leaf teas are water‐soluble, to develop tea products with health benefits from blueberry leaves would be practical and significant.

## 4 CONCLUSIONS

The nonfermented green tea, semifermented oolong tea, and fully fermented red tea were manufactured from the fresh blueberry leaves, and their chemical constituents as well as antioxidant activities in vitro were compared in the present study. The composition and content of main components, including amino acids, phenolics/flavonoids, and volatiles, were found to vary significantly in the different types of teas. With minimum processing, the GT possessed the highest contents of phenolics and flavonoids, which decreased dramatically in the OT and RT, showing an inverse correlation with the fermentation degree. On the contrary, fermentation process could lead to the formation of new volatile compounds and fermented tea flavor in the OT and RT. The three types of blueberry leaf teas all showed potent antioxidant activities. The antioxidant activities decreased in the order of GT > OT>RT, in a fermentation degree‐dependent manner. The findings of this study might provide a promising insight on the utilization of blueberry leaves and exploration of novel tea varieties as functional food sources.

## CONFLICTS OF INTEREST

The authors have declared no conflict of interest.

## ETHICAL STATEMENT

The authors declare that this study did not involve human or animal subjects and human and animal testing are unnecessary in our study.
